# Mouse Testicular Cell Type-Specific Antiviral Response against Mumps Virus Replication

**DOI:** 10.3389/fimmu.2017.00117

**Published:** 2017-02-10

**Authors:** Han Wu, Xiang Zhao, Fei Wang, Qian Jiang, Lili Shi, Maolei Gong, Weihua Liu, Bo Gao, Chengyi Song, Qihan Li, Yongmei Chen, Daishu Han

**Affiliations:** ^1^School of Basic Medicine, Peking Union Medical College, Institute of Basic Medical Sciences, Chinese Academy of Medical Sciences, Beijing, China; ^2^Joint International Research Laboratory of Agriculture and Agri-Product Safety, College of Animal Science and Technology, Institute of Epigenetics and Epigenomics, Yangzhou University, Yangzhou, China; ^3^Institute of Medical Biology, Chinese Academy of Medical Sciences, Kunming, China

**Keywords:** mumps virus, testicular cell, viral replication, innate antiviral response, autophagy

## Abstract

Mumps virus (MuV) infection has high tropism to the testis and usually leads to orchitis, an etiological factor in male infertility. However, MuV replication in testicular cells and the cellular antiviral responses against MuV are not fully understood. The present study showed that MuV infected the majority of testicular cells, including Leydig cells (LC), testicular macrophages, Sertoli cells (SC), and male germ cells (GC). MuV was replicated at relatively high efficiencies in SC compared with LC and testicular macrophages. In contrast, MuV did not replicate in male GC. Notably, testicular cells exhibited different innate antiviral responses against MuV replication. We showed that interferon β (IFN-β) inhibited MuV replication in LC, macrophages, and SC, which were associated with the upregulation of major antiviral proteins. We provided primary evidence that autophagy plays a role in blocking MuV replication in male GC. Autophagy was also involved in limiting MuV replication in testicular macrophages but not in Leydig and SC. These findings indicate the involvement of the innate defense against MuV replication in testicular cells.

## Introduction

Mumps virus (MuV) infection usually causes orchitis and may result in male infertility (1). Mumps orchitis is associated with direct MuV infection in the testis. Understanding the innate antiviral response against MuV replication in testicular cells can aid in the development of therapeutic strategies for mumps orchitis.

Mumps virus is an enveloped negative-sense RNA virus (2). An evident manifestation of MuV infection is painful parotitis, a contagious disease that occurs worldwide. MuV infection may also result in the inflammation of several other organs, including encephalitis, meningitis, myocarditis, pancreatitis, nephritis, and orchitis (3). Notably, mumps orchitis is the most common extra-salivary gland inflammation caused by MuV infection (4). The recovery of MuV from the testis and the semen of mumps orchitis patients suggest that a direct MuV infection in the testis is associated with diseases (5, 6). However, MuV infection and replication in testicular cells remain elusive.

Although humans are believed to be the only natural hosts, MuV also infects animal cells *via* its receptor, sialic acid, which is present on the surface of most animal cells (7). Viral replication in infected cells is controlled by cellular innate antiviral response. Type 1 interferon (IFN-α and IFN-β) production is a universal mechanism of the host’s defense against viral infection (8). IFN-α and IFN-β can be produced by most cell types in response to viral infection through the activation of pattern recognition receptors (PRRs) (9). Type 1 IFNs induce the expression of various antiviral proteins such as IFN-stimulated gene 15 (ISG15), 2′-5′-oligoadenylate synthetase 1 (OAS1), and Mx GTPase 1 (MX1), thereby inhibiting viral replication and degrading viral nucleic acids in infected cells (10). Type 1 IFNs also promote the host’s adaptive immune response against viral infection (11). Recently, we showed that MuV infection induced IFN-α and IFN-β production in Sertoli and Leydig cells (LC) (12). However, the role of IFNs in the testicular cell defense against MuV has yet to be clarified.

Autophagy is a conserved lysosome-dependent degradation pathway that breaks down dysfunctional organelles and large protein aggregates, which are involved in multiple pathophysiological conditions (13). Autophagy is also an intracellular innate defense mechanism against microbial infection by directly degrading microbes such as viruses, bacteria, and protozoa that invade cells (14, 15). The autophagy pathway is tightly regulated by a panel of autophagy-related proteins. Beclin-1 and microtubule-associated protein light chain 3 (LC3) are two critical autophagy-related proteins. Beclin-1 orchestrates different stages of autophagosome assembly (16), and LC3 is a hallmark of autophagosomal maturation (17).

The mammalian testis is a remarkable immumoprivileged organ necessary for protecting immunogenic germ cells (GC) from detrimental immune responses (18). To overcome immunoprivileged environment, the testis adapts local innate defense system against microbial infections (19). Although PRR-initiated innate immune responses to pathogen-associated molecules in testicular cells have been revealed, the functions of the innate immune responses in the testicular cell defense against live natural microbes need to be formally demonstrated. Moreover, male GC are equipped with autophagic machinery (20). The potential role of autophagy in the testicular cell defense against microbial infections has yet to be investigated. The present study elucidated the cell type-specific roles of IFN response and autophagy in mouse testicular cell defense against MuV replication.

## Materials and Methods

### Mice

C57BL/6 mice were purchased from the Laboratory Animal Center of Peking Union Medical College. The mice were maintained in a specific pathogen-free facility with 12/12 h light/dark cycle and were provided with food and water *ad libitum*. All mice were handled in compliance with the Guidelines [permit number: SCXK (Jing) 2007-0001] for the Care and Use of Laboratory Animals established by the Chinese Council on Animal Care (Beijing, China). The protocol was approved by Animal Care and Use Committee of Institute of Basic Medical Sciences in China.

### Antibodies and Major Reagents

Mouse anti-MuV nucleoprotein (MuV-NP) (ab9876), rat anti-F4/80 (ab6640), and anti-IFN-β (ab24324) mAbs were purchased from Abcam (Cambridge, UK). Rabbit anti-MX1 (sc-50509), anti-LHR (sc-25828), anti-Wilms tumor nuclear protein 1 (WT1) (sc-192) polyclonal Abs, goat anti-Beclin-1 (sc-10086) polyclonal Abs, and mouse anti-OAS1 (sc-365072) mAb were purchased from Santa Cruz Biotechnology (Santa Cruz, CA, USA). Rabbit anti-ISG15 (#2743) polyclonal Abs and anti-LC3 (#4599) mAb were purchased from Cell Signaling Technology (Beverly, MA, USA). Mouse anti-β-actin (A5316) mAbs were purchased from Sigma-Aldrich (St. Louis, MO, USA). 4′, 6′-diamidino-2-phenylindole (DAPI), horseradish-peroxidase (HRP)-, and fluorescein isothiocyanate (FITC)-conjugated secondary Abs were purchased from Zhongshan Biotechnology (Beijing, China). Mouse recombinant IFN-β (no. 19032) was purchased from Sigma-Aldrich. 3-methyladenine (3-MA) (tlrl-3ma) was purchased from InvivoGen (San Diego, CA, USA).

### Cell Isolation

Testicular cells were isolated from 4-week-old C57BL/6 mice based on previously described procedures (21). The testes of three mice were decapsulated and incubated with 0.5 mg/mL collagenase type 1 (Sigma-Aldrich) in F12/DMEM (Life Technologies, Grand Island, NY, USA) at room temperature for 15 min with gentle oscillation. The suspensions were filtered through 80-µm copper mesh to separate interstitial cells from the seminiferous tubules. The interstitial cells were cultured in F12/DMEM supplemented with 100 U/mL penicillin, 100 mg/mL streptomycin, and 10% fetal calf serum (FCS; Life Technologies). After 24 h, LC were detached by treatment with 0.125% trypsin for 5 min. Testicular macrophages remained attached on culture dishes after the trypsin treatment. The purities of LC and macrophages were assessed by immunostaining for luteinizing hormone receptor (LHR), a marker of LC (22), and for F4/80, a marker of macrophages (23).

The seminiferous tubules were incubated with 0.5 mg/mL collagenase type 1 for 15 min at room temperature to remove peritubular myoid cells. The tubules were cut into small pieces of approximately 1 mm and incubated with 0.5 mg/mL hyaluronidase (Sigma-Aldrich) at room temperature for 10 min with gentle pipetting to dissociate GC from Sertoli cells (SC). Suspensions were cultured with F12/DMEM at 32°C for 6 h. The GC were recovered by collecting non-adherent cells. The germ cell purity was assessed based on cell nuclear morphology after staining with DAPI (24). SC were cultured for additional 24 h and then treated with a hypotonic solution (20 mM Tris, pH 7.4) for 1 min to remove adhering GC. The purity of SC was assessed by immunofluorescence staining of WT1, a marker of SC (25). Testicular cells were incubated in a humidified atmosphere containing 5% CO_2_ at 32°C.

### MuV Infection

Mumps virus (SP-A strain) was isolated from mumps patients (26) and obtained from the Institute of Medical Biology, Chinese Academy of Medical Sciences (Kunming, China). MuV was amplified and titrated in Vero cells. MuV preparations were diluted in 1× PBS at a density of 1 × 10^9^ plaque forming unit/mL and stored at −80°C. For infection, testicular cells were inoculated with MuV for 2 h. Cells were treated with 0.25% trypsin for 5 min at room temperature to remove MuV attaching on cell surfaces. After washing thrice with PBS, cells were cultured in F12/DMEM supplemented with 100 U/mL penicillin, 100 mg/mL streptomycin, and 10% FCS.

### Plaque Assay

Mumps virus titers were assessed using plaque assay in Vero cells. Briefly, Vero cells were cultured in 6-well plates at 2 × 10^5^ cells/well and infected with a serial dilution of MuV for 1 h. Cells were washed three times with PBS and then cultured in DMEM containing 2% FCS and 1.5% methylcellulose. After 1 week, cells were stained with 1% crystal violet solution (Sigma-Aldrich) in accordance with the manufacturer’s instructions. Plaques were counted and represented MuV particles.

### MTT Assay

Cell viability was assessed using an MTT Assay Kit (ATCC, Manassas, VA, USA) in accordance with the manufacturer’s instructions. In brief, cells were cultured in 96-well microplates at a density of 2 × 10^4^ cells/well. After treatments with MuV and 3-MA, cells were incubated with 10 µL of MTT solution for 2 h. Then, 100 µL of detergent reagent, which was included in the kit, was added to each well. Absorbance at 570 nm was determined with a microplate reader (BioTek, Winooski, VT, USA). The percentage of the absorbance value versus the control value represents cell viability.

### Real-time Quantitative RT-PCR (qRT-PCR)

Total RNA was extracted with Trizol™ reagent (Invitrogen, Carlsbad, CA, USA) in accordance with the manufacturer’s instructions. RNA was treated with RNase-free DNase 1 (Invitrogen) to eliminate potential DNA contaminants. RNA (1 µg) was reverse transcribed into cDNA in a 20 µL reaction mixture containing 2.5 µM random hexamers, 2 µM dNTPs, and 200 U Moloney murine leukemia virus reverse transcriptase (Promega, Madison, WI, USA). PCR was performed using a Power SYBR^®^ Green PCR Master Mix Kit (Applied Biosystems, Foster City, CA, USA) on an ABI PRISM 7300 real-time cycler (Applied Biosystems). Relative mRNA levels were determined by the comparative 2^−∆∆CT^ method as described in the Applied Biosystems User Bulletin No. 2 (P/N 4303859) (27). The primer sequences are listed in Table 1.

**Table 1:** **Primers used for real-time PCR**.

Target genes	Primer pairs (5′–3′)	
	Forward	Reverse
MuV nucleoprotein	TCAGATCAATCGCATCGGGG	CTTGCGACTGTGCGTTTTGA
IFN-stimulated gene 15	CCAGTCTCTGACTGTGAGAGC	GCATCACTGTGCTGCTGGGAC
2′-5′-oligoadenylate synthetase 1	ATTACCTCCTTCCCGACACC	CAAACTCCACCTCCTGATGC
Mx GTPase 1	GACCATAGGGGTCTTGACCAA	AGACTTGCTCTTTCTGAAAAGCC
Beclin-1	GAGCCATTTATTGAAACTCGCCA	CCTCCCCGATCAGAGTGAA
LC3a	GCGAGTTGGTCAAGATCATCC	ATGTCAGCGATGGGTGTGGA
β-actin	GAAATCGTGCGTGACATCAAAG	TGTAGTTTCATGGATGCCACAG

### Western Blot Analysis

The cells were lysed with a lysis buffer (Applygen Technologies Inc., Beijing, China), and the protein concentrations were determined with a Bicinchoninic Acid Protein Assay Kit (Pierce Biotechnology, Rockford, IL, USA). Proteins (20 μg/well) were separated on 12% SDS-PAGE gel and then electrotransferred onto polyvinyl difluoride membranes (Millipore, Bedford, MA, USA). The membranes were blocked with Tris-buffered saline (TBS, pH 7.4) containing 5% non-fat milk at room temperature for 1 h and then incubated with the primary antibodies overnight at 4°C. The membranes were washed twice with TBS containing 0.1% Tween-20 and then incubated with the HRP-conjugated secondary antibodies at room temperature for 1 h. Ag/Ab complexes were visualized with an enhanced Chemiluminescence Detection Kit (Zhongshan Biotechnology Co.). β-Actin was used as the loading control.

### Immunohistochemistry and Immunofluorescence Staining

The testes were fixed in Bouin’s solution for 24 h and then embedded in paraffin. Sections (5 µm in thickness) were cut using rotary microtome Reichert 820 HistoSTAT (Reichert Technologies, NY, USA). After dewaxing process, the sections were incubated with 3% H_2_O_2_ in PBS for 15 min at room temperature to inhibit endogenous peroxidase activity. The slides were soaked in citrate buffer and microwave heated at 100°C for 10 min to retrieve the antigens. After blocking with 5% normal goat sera in PBS for 1 h at room temperature, the sections were incubated with primary antibodies overnight at 4°C. After washing twice with PBS, the sections were incubated with appropriate FITC- or HRP-conjugated secondary antibodies (Zhongshan Biotechnology Co.) at room temperature for 30 min. HRP activity was visualized using the diaminobenzidine method. Negative controls were incubated with appropriate pre-immune animal (rabbit, rat, or mouse) sera instead of primary antibodies. The sections were counterstained with DAPI in immunofluorescence staining or with hematoxylin in immunohistochemical staining and then mounted with neutral balsam (Zhongshan Biotechnology Co.) for observation.

For indirect immunofluorescence staining, cells were fixed with −20°C pre-cooled methanol for 3 min and then permeabilized with 0.2% Triton X-100 in PBS for 15 min. The cells were blocked with 5% normal goat sera for 30 min at room temperature and then incubated with primary antibodies at 37°C for 90 min. The cells were washed twice with PBS and incubated with appropriate FITC-conjugated secondary antibodies (Zhongshan Biotechnology Co.) for 30 min at 37°C. The slides were mounted with antifade mounting medium (Zhongshan Biotechnology Co.) and observed under a fluorescence microscope BX-51 (Olympus, Tokyo, Japan).

### Statistical Analysis

Data were presented as the mean ± SEM of at least three independent experiments. Statistical difference between two groups was determined using Student’s *t*-test. One-way ANOVA with Bonferroni’s (selected pairs) *post hoc* test was used for multiple comparisons. The calculations were performed with SPSS version 13.0 (SPSS Inc., Chicago, IL, USA). *P* < 0.05 was considered statistically significant.

## Results

### MuV Infection of Testicular Cells

**Figure 1:**
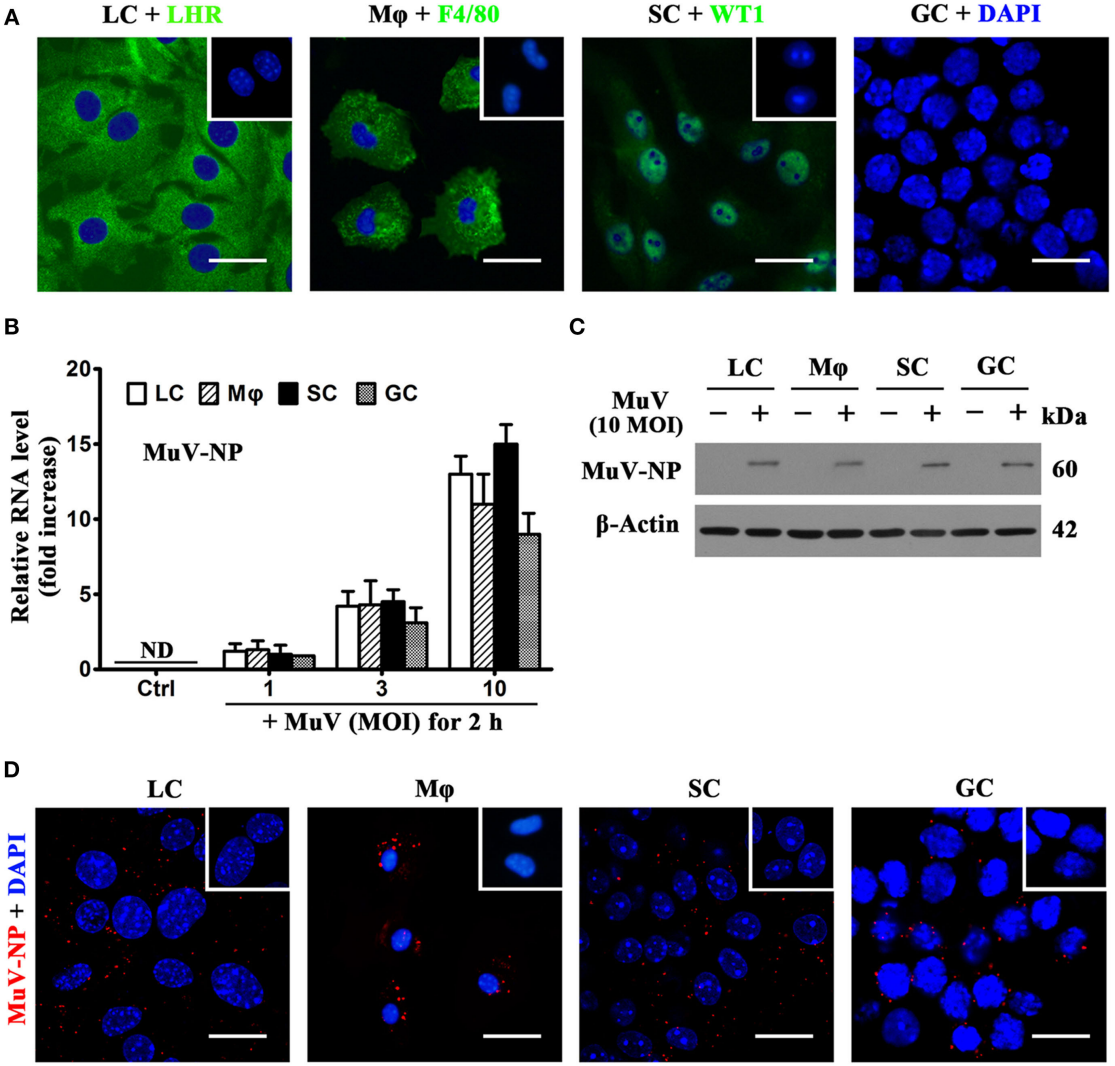
**Identification and mumps virus (MuV) infection of testicular cells**. Major testicular cells, including Leydig cells (LC), macrophages (Mφ), Sertoli cells (SC), and germ cells (GC), were isolated from 4-week-old C57BL/6 male mice. **(A)** Identification of cell purity. Purities of somatic cells were determined using indirect immunofluorescence staining of cell markers, luteinizing hormone receptor (LHR) for LC, F4/80 for Mφ, and Wilms tumor nuclear protein 1 (WT1) for SC. Insets in the upper right corners represent negative controls (Ctrl), in which pre-immune rabbit or rat sera served as primary antibodies, respectively. GC purity was assessed by nuclear morphologies after staining with 4′, 6′-diamidino-2-phenylindole (DAPI). **(B)** MuV nuclear protein (MuV-NP) RNA level in infected cells. Testicular cells were infected with the indicated multiplicity of infection (MOI) of MuV for 2 h. Total RNAs were extracted, and relative RNA levels of MuV-NP were determined using quantitative real-time PCR (qRT-PCR). Cells without MuV infection served as Ctrl. ND, not detectable. **(C)** Protein levels of MuV-NP. Cells were uninfected or infected with 10 MOI MuV for 2 h, and MuV-NP was determined using Western blot analysis. β-Actin was used as the loading control. **(D)** MuV particles. Testicular cells were infected with 10 MOI MuV for 2 h. Intracellular MuV was assessed using immunofluorescence staining of MuV-NP. Insets in the upper right corners represent negative Ctrl, in which pre-immune mouse sera served as primary antibodies. Images represent at least three independent experiments. Data are presented as the means ± SEM of three experiments. Scale bars, 20 µm.

To examine MuV infection of testicular cells, including LC, testicular macrophages, SC, and GC were isolated from 4-week-old C57BL/6 male mice. Cell purity was assessed using indirect immunofluorescence staining of LHR for LC, F4/80 for macrophages, and WT1 for SC (Figure 1A). Germ cell purity was assessed based on nuclear morphology after staining with DAPI (Figure 1A, right panel). The purity of LC and macrophages was >92%, and the purity of Sertoli and GC was >95%. To determine MuV infection in testicular cells, the levels of genomic MuV-NP RNA were examined by real-time qRT-PCR at 2 h after infection with different doses of MuV (Figure 1B). MuV-NP RNA was undetectable in control cells without MuV infection. Western blot analysis results showed that the MuV-NP protein levels in the testicular cells at 2 h after infection with 10 multiplicity of infection (MOI) MuV (Figure 1C). MuV-NP proteins in testicular cells were confirmed by immunofluorescence staining at 2 h after infection with 10 MOI MuV (Figure 1D). These findings suggest that MuV infects major testicular cells.

### MuV Replication in Testicular Cells

**Figure 2:**
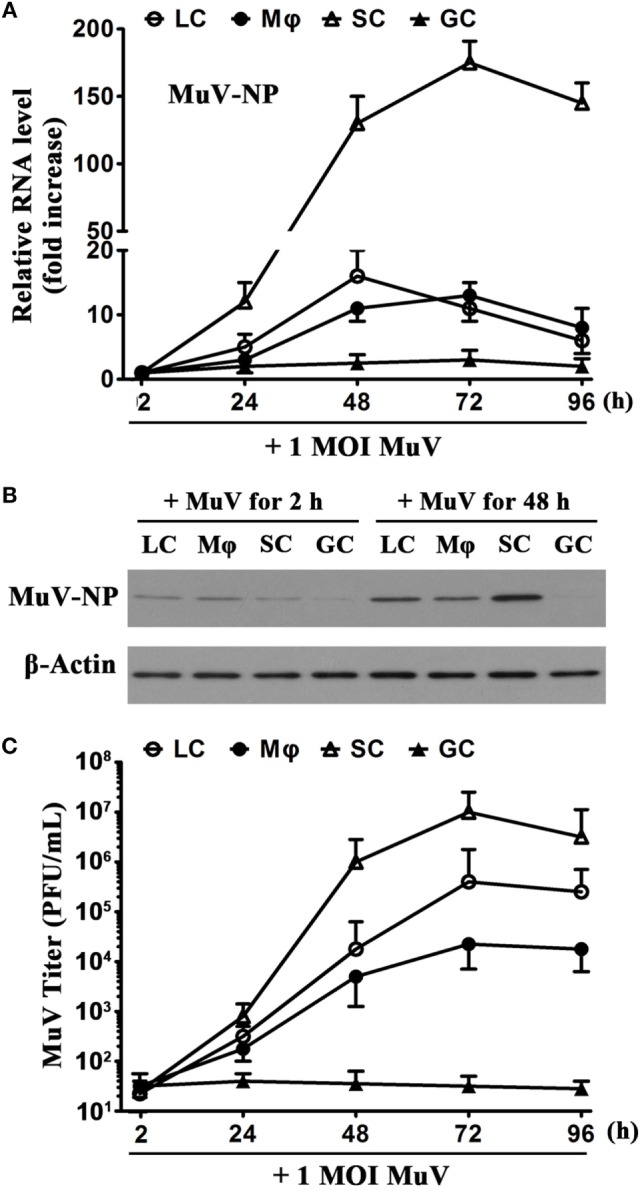
**Mumps virus (MuV) replication**. **(A)** Time-dependent MuV replication in testicular cells. Cells were seeded in 6-well plates at a density of 2 × 10^5^/well and infected with one multiplicity of infection (MOI) MuV for specific durations. Relative RNA levels of MuV-NP were determined using real-time qRT-PCR. **(B)** Protein levels of MuV-NP. Cells were infected with 1 MOI MuV. MuV-NP protein levels in testicular cells at 2 and 48 h after infection were determined using Western blot analysis. **(C)** MuV titers. Cells were infected as described in **(A)**. MuV titers in the culture media were analyzed using plaque assay. PFU, plaque forming unit. The limit of detection was 10. Images represent at least three independent experiments. Date are presented as the means ± SEM of three experiments.

To evaluate MuV replication, we examined MuV-NP expression in testicular cells and viral titers in culture media. Real-time qRT-PCR analysis showed that MuV-NP RNA levels were increased in SC in a time-dependent manner after infection with one MOI MuV (Figure 2A). MuV-NP RNA levels were also evidently increased in LC and macrophages. Notably, the peak MuV-NP RNA level in SC was about 10-fold higher than that in LC and macrophages. In contrast, the level of MuV-NP RNA in male GC did not significantly increase. Western blot analysis also confirmed that the MuV-NP protein levels were apparently increased in testicular somatic cells but not in GC at 48 h post MuV infection (Figure 2B). Plaque forming assay results showed that MuV titers in the media of testicular somatic cells were increased in a time-dependent manner (Figure 2C). MuV titers were not increased in the media of male GC. These observations indicated that MuV significantly replicate in testicular somatic cells but not in male GC.

### Inhibition of MuV Replication by IFN-β

**Figure 3:**
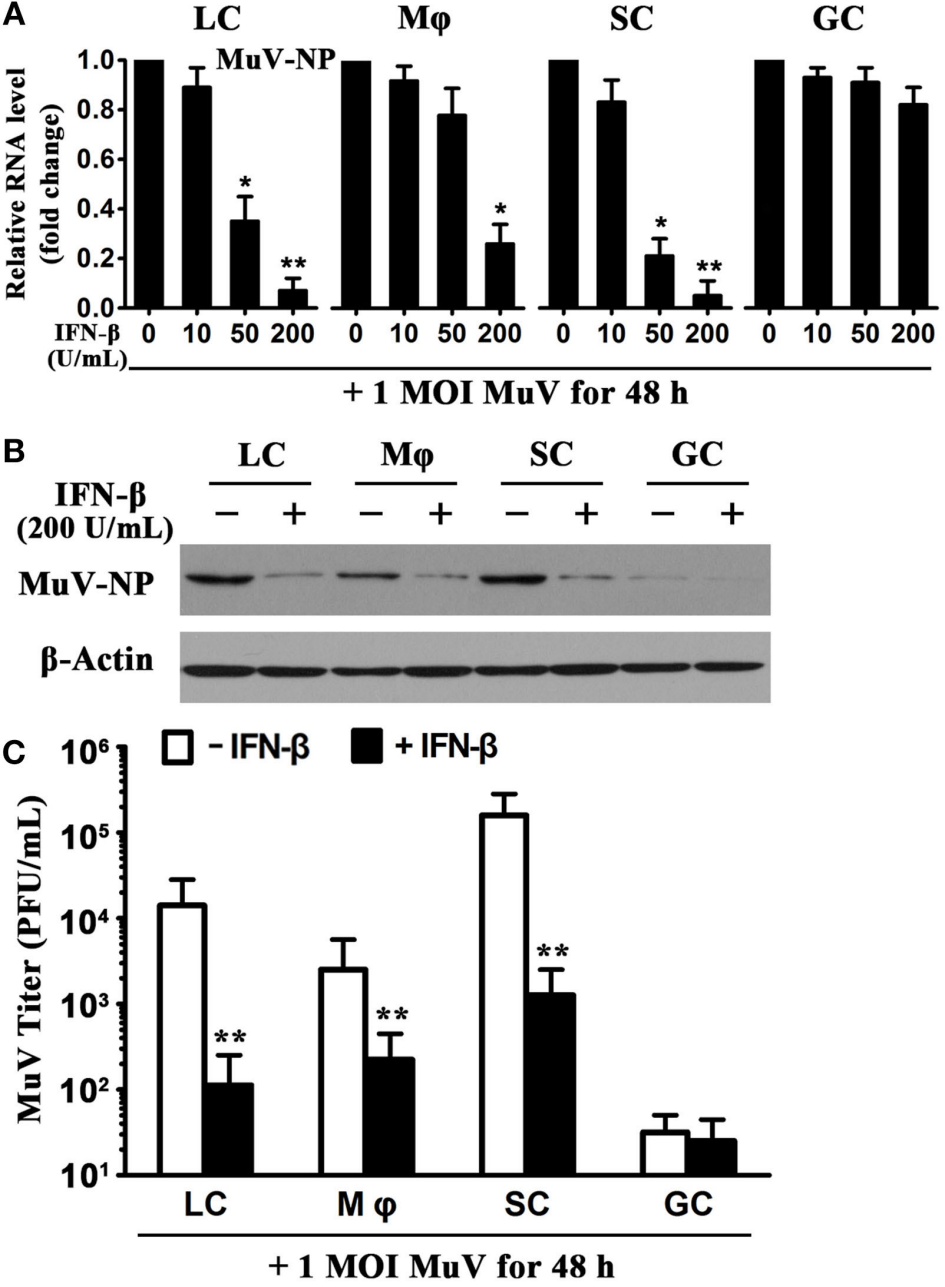
**Inhibition of mumps virus (MuV) replication by interferon β (IFN-β)**. **(A)** A dose-dependent IFN-β inhibition of MuV replication. Testicular cells were seeded in 6-well plates at a density of 2 × 10^5^ cells/well and infected with one multiplicity of infection (MOI) MuV in the presence of the indicated doses of IFN-β. After 48 h, total RNAs were extracted, and relative RNA levels of MuV-NP were determined using real-time qRT-PCR. **(B)** Protein levels of MuV-NP. Cells were infected with 1 MOI MuV in the absence and presence of 200 U/mL IFN-β. At 48 h after MuV infection, MuV-NP was determined using Western blot analysis. **(C)** MuV titers. Cells were treated as described in **(B)**. MuV titers in culture media were analyzed using plaque forming assay. The limit of detection was 10. Images represent at least three independent experiments. Data are presented as the means ± SEM of three experiments (*p < 0.05, **p < 0.01).

Given that IFN-β is a critical antiviral cytokine (28), we examined the effect of recombinant IFN-β on MuV replication in testicular cells. The cells were infected with one MOI MuV in the presence of different doses of IFN-β. Real-time qRT-PCR analysis showed that the presence of 200 U/mL IFN-β significantly reduced the MuV-NP RNA levels in LC, macrophages, and SC at 48 h after MuV infection (Figure 3A). In contrast, IFN-β did not affect the MuV-NP RNA levels of male GC (Figure 3A, right panel). Western blot analysis confirmed that IFN-β evidently decreased the MuV-NP protein levels in Leydig, macrophages, and SC (Figure 3B). MuV-NP protein levels in male GC were not altered by IFN-β. Plaque forming assay results showed that MuV titers in the media of testicular somatic cells were significantly reduced by IFN-β at 48 h after MuV infection (Figure 3C). These results imply that exogenous IFN-β significantly inhibits MuV replication in testicular somatic cells but not GC.

### Induction of Antiviral Proteins by IFN-β

**Figure 4:**
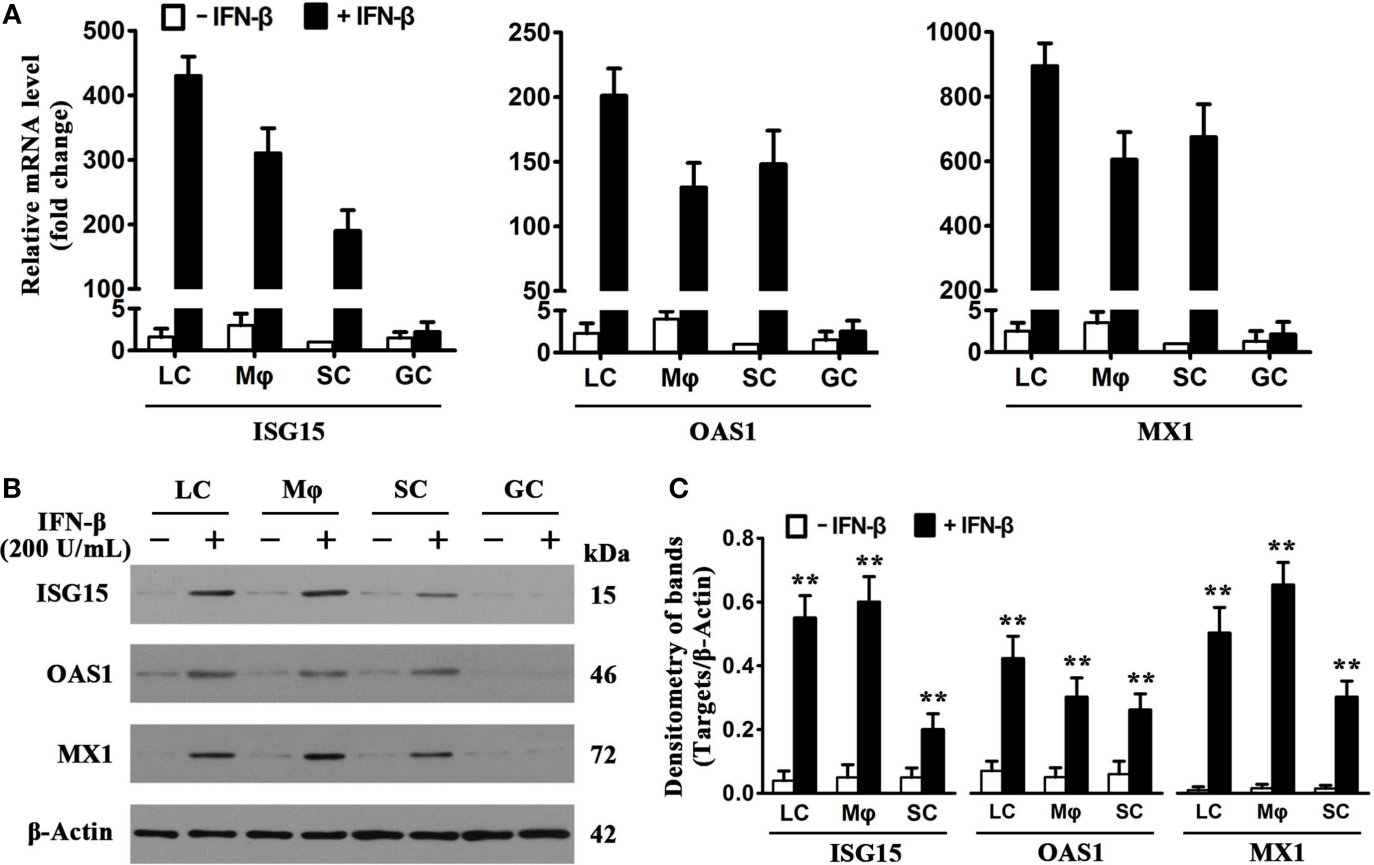
**Induction of antiviral proteins by interferon β (IFN-β)**. **(A)** mRNA levels of antiviral proteins. Testicular cells were cultured in the absence and presence of 200 U/mL IFN-β for 8 h. Total RNA was extracted, and relative mRNA levels of major antiviral proteins, including IFN-stimulated gene 15 (ISG15), 2′-5′-oligoadenylate synthetase 1 (OAS1), and Mx GTPase 1 (MX1), were determined using real-time qRT-PCR. The lowest mRNA levels of the antiviral proteins were set as “1.” Fold changes normalizing to 1 were presented. Data are presented as the means ± SEM of three independent experiments. **(B)** Protein levels of the antiviral proteins. Testicular cells were cultured in the absence and the presence of 200 U/mL IFN-β for 24 h, cell lysates were subjected to Western blot analysis. **(C)** Antiviral protein levels were quantitatively analyzed by measuring densitometry of bands in Western blots. Images represent at least three independent experiments. Data are presented as the means ± SEM of three experiments (*p < 0.05, **p < 0.01).

Considering that IFN-inducible antiviral proteins play crucial roles in restricting viral replication in infected cells (8), we examined the expression of major antiviral proteins, including ISG15, OAS1, and MX1, in testicular cells in response to IFN-β treatment. Real-time qRT-PCR results (Figure 4A) showed that the presence of 200 U/mL IFN-β dramatically increased the mRNA levels of ISG15 (left panel), OAS1 (middle panel), and MX1 (right panel) in LC, macrophages, and SC at 8 h post-infection. IFN-β insignificantly induced the antiviral protein mRNA levels of male GC. Western blot analysis confirmed that the antiviral protein levels were significantly upregulated in the testicular somatic cells 24 h after the presence of IFN-β (Figures 4B,C). In contrast, IFN-β did not increase the levels of the antiviral proteins in male GC.

### Role of MuV-Induced IFN-β in Antiviral Response

**Figure 5:**
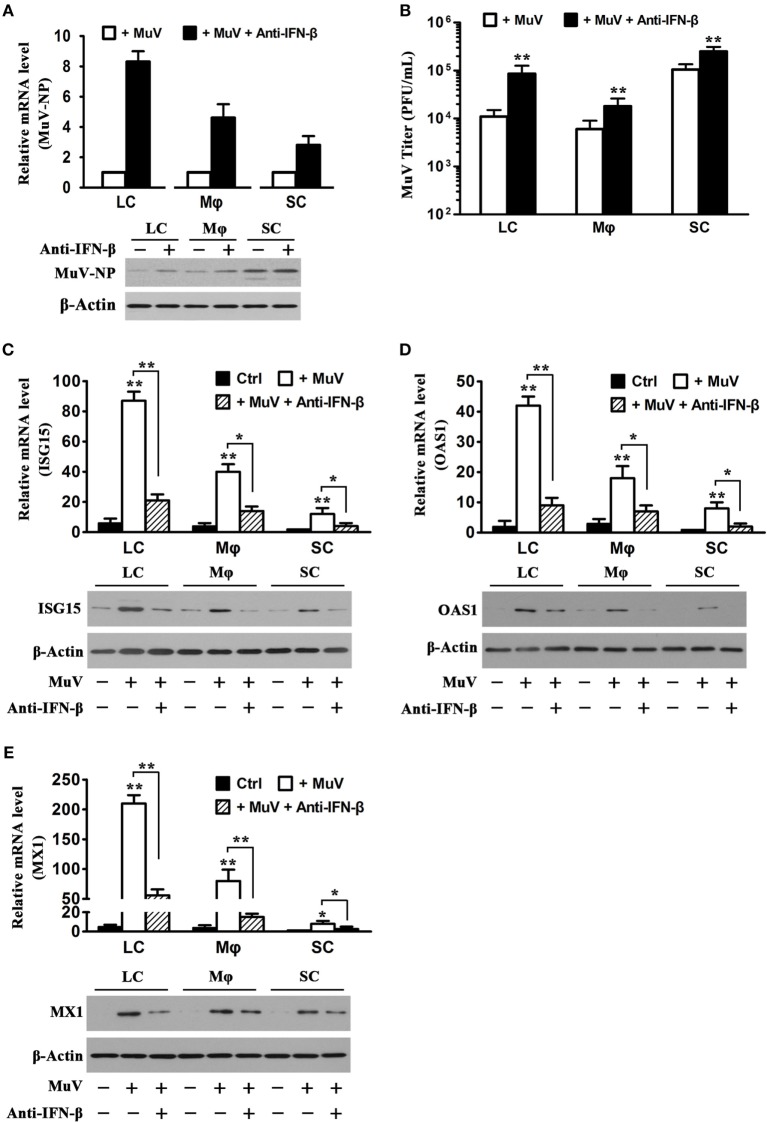
**Role of interferon β (IFN-β) in mumps virus (MuV)-induced antiviral response**. **(A)** Effect of anti-IFN-β antibodies. LC, Mφ, and SC were infected with one multiplicity of infection (MOI) MuV or infected with 1 MOI MuV in the presence of 5 µg/mL anti-IFN-β antibodies for 48 h. MuV-NP RNA (upper panel) and protein (lower panel) levels were determined by real-time qRT-PCR and Western blot analysis, respectively. **(B)** MuV titers. Testicular cells were treated as described in **(A)**. MuV titers in cultured media were determined using plaque forming assay. **(C–E)** Expression of antiviral proteins. Testicular cells were treated as described in **(A)**. The cells without treatment served as controls (Ctrl). The mRNA (upper panel) and protein (lower panel) levels of IFN-stimulated gene 15 (ISG15) **(C)**, OAS1 **(D)**, and Mx GTPase 1 (MX1) **(E)** were determined by real-time qRT-PCR and Western blot analysis. Images of Western blot represent at least three independent experiments. Data are presented as the means ± SEM of three experiments.

Given that MuV induces IFN-β production in Sertoli and LC (12), we speculated that MuV-induced IFN-β is involved in antiviral responses in testicular somatic cells. Therefore, we examined the effects of neutralizing antibodies against IFN-β on MuV replication. The presence of 5 µg/mL anti-IFN-β antibodies evidently increased MuV-NP RNA (Figure 5A, upper panel) and protein (low panel) levels in LC and macrophages at 48 h after infection with 1 MOI MuV. The increases in MuV-NP RNA and protein levels by anti-IFN-β antibodies were less evident in SC. Accordingly, the anti-IFN-β antibodies significantly increased MuV titers in the culture media (Figure 5B). The mRNA (upper panel) and protein (low panels) levels of ISG15 were remarkably increased in the testicular cells at 48 h after MuV infection (Figure 5C). The MuV-induced ISG15 levels were significantly reduced in the presence of anti-IFN-β antibodies. Similarly, MuV significantly increased OAS1 (Figure 5D) and MX1 (Figure 5E) levels, which were reduced by the anti-IFN-β antibodies.

### Autophagic Machinery in Testicular Cells

**Figure 6:**
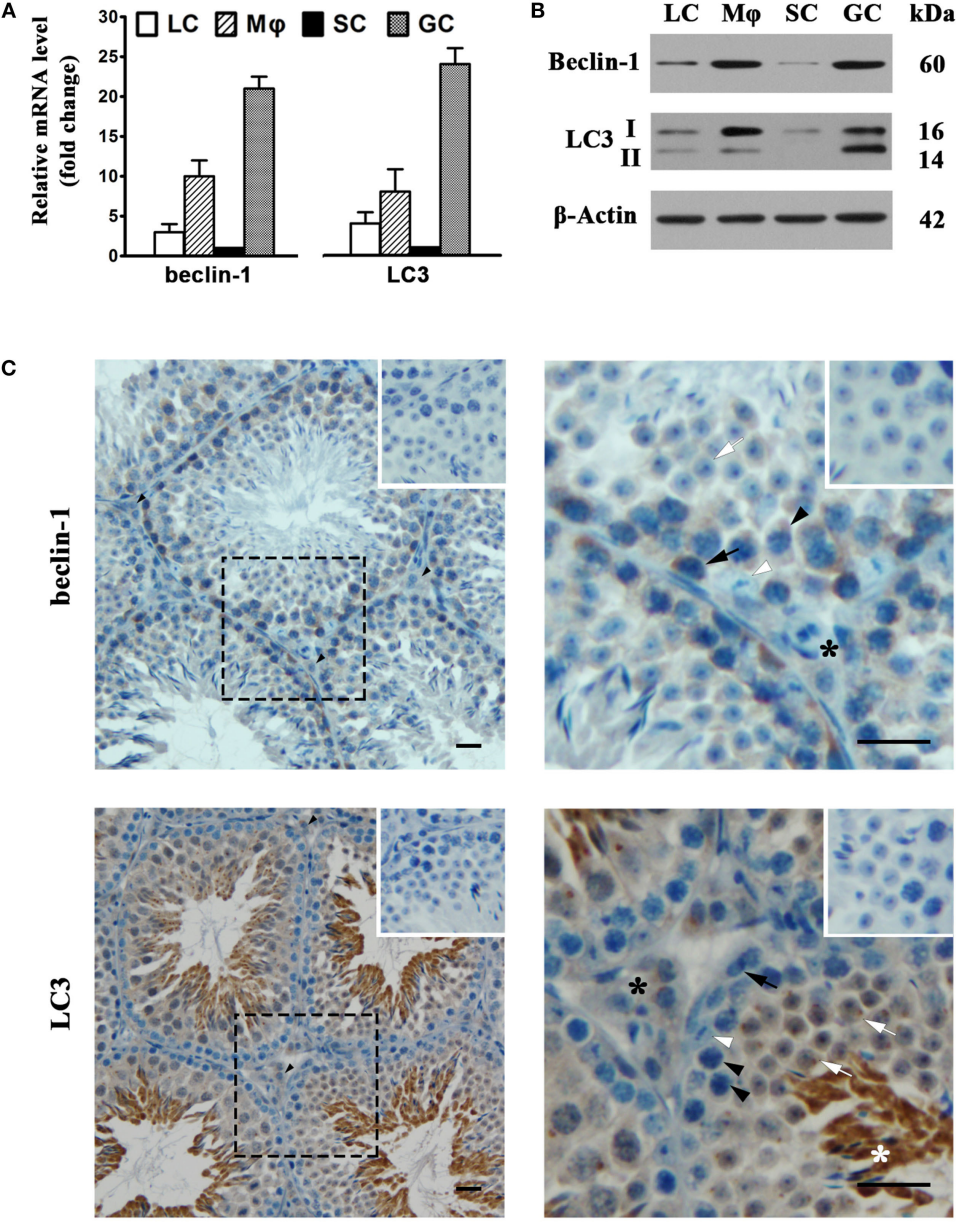
**Autophagy-related gene expression**. **(A)** The mRNA levels of beclin-1 and light chain 3 (LC3). Testicular cells were isolated from 4-week-old mice. Relative mRNA levels of beclin-1 and LC3 were determined using real-time qRT-PCR. **(B)** Protein levels of beclin-1 and LC3. Testicular cell lysates were subjected to Western blot analysis. β-Actin was used as loading control. **(C)** Cellular distribution of beclin-1 and LC3. The paraffin sections of the testes from 5-week-old mice were subjected to immunohistochemistry analysis to locate beclin-1 (upper panels) and LC3 (low panels) using specific antibodies. Insets in the upper right corners represent negative controls, in which pre-immune rabbit sera served as primary antibodies. Black arrows, spermatogonia; black arrowheads, spermatocytes; white arrows, round spermatids; white arrowheads, Sertoli cells (SC); black asterisks, interstitial cells; white asterisks, elongating spermatids. Data are presented as the means ± SEM of three experiments. Images represent at least three independent experiments. Scale bars, 20 µm.

To assess autophagy in testicular cells, we examined the expression of two major autophagy-related proteins, namely, beclin-1 and LC3. Real-time qRT-PCR results showed that male GC abundantly expressed beclin-1 and LC3 at mRNA levels (Figure 6A). The mRNA levels of beclin-1 and LC3 were relatively low in Leydig and SC. Beclin-1 and LC3 mRNAs were also evident in macrophages. Western blot analysis also detected the protein expression of beclin-1 and LC3 in testicular cells (Figure 6B). The most abundant type II LC3 (LC3-II) was detected in male GC, suggesting that these cells are capable of autophagy. Moreover, the beclin-1 and LC3 proteins were evidently detected in macrophages, suggesting that testicular macrophages are also well equipped with autophagic machinery. In contrast, the beclin-1 and LC3 were faintly detected in Leydig and SC. Immunohistochemistry analysis confirmed that beclin-1 (Figure 6C, upper panels) was localized in spermatogonia (black arrows), spermatocytes (black arrowheads), round spermatids (white arrows), and interstitial cells (black asterisks). In contrast, beclin-1 was not evident in SC (white arrowheads). LC3 was predominantly localized to round and elongating spermatids (white asterisks) (Figure 6C, lower panel). LC3 was also detected in interstitial cells.

### Role of Autophagy in Limiting MuV Replication

**Figure 7:**
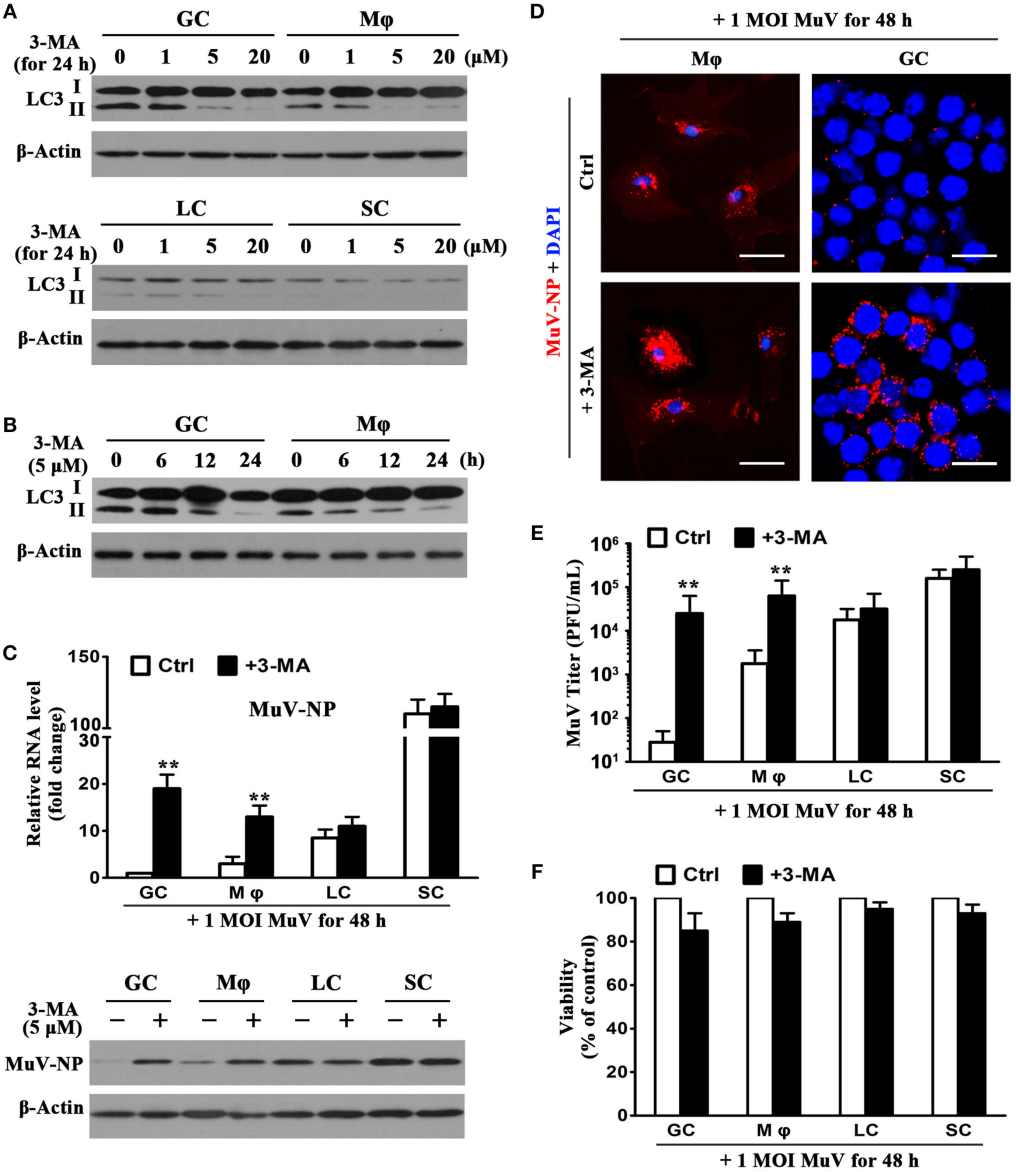
**Role of autophagy in limiting mumps virus (MuV) replication**. **(A)** Inhibition of autophagy by 3-methyladenine (3-MA). Germ cells (GC) and macrophages (Mφ) (upper panels), as well as Leydig cells (LC) and Sertoli cells (SC) (low panels), were treated with different doses of 3-MA for 24 h. LC3, including LC3-I and LC3-II, were determined by Western blot analysis. **(B)** A time-dependent effect of 3-MA on LC3 expression. GC and Mφ were treated with 5 µM 3-MA for specific durations. LC3 levels were analyzed by Western blot analysis. **(C)** Effect of 3-MA on MuV-NP levels. Testicular cells were infected with one multiplicity of infection (MOI) MuV in the absence (Ctrl) and presence of 5 µM 3-MA. After 48 h, MuV-NP RNA levels were determined using real-time qRT-PCR (upper panel), and its protein levels were assessed by Western blot analysis. **(D)** Intracellular MuV particles. Mφ (left panels), and GC (right panels) were treated as described in **(C)** for 48 h. MuV particles were detected by immunofluorescence staining using antibodies against MuV-NP. Cell nuclei were visualized using staining with DAPI. Scale bars, 20 µm. **(E)** MuV titers. Cells were treated as described in **(C)** for 48 h. MuV titers in media were determined using plaque forming assay. The limit of detection was 10. **(F)** Cell viability. Cells were treated as described in **(C)** for 48 h. Cell viability was assessed by MTT assay. Images represent at least three independent experiments. Data are presented as the means ± SEM of three experiments (**p < 0.01).

The effect of 3-MA (an autophagy inhibitor) on MuV replication in testicular cells was examined to determine the role of autophagy in antiviral ability. The presence of 3-MA for 24 h inhibited the LC3-II level of GC and macrophages in a dose-dependent manner (Figure 7A, upper panels). 3-MA at concentrations of 5 and 20 µM remarkably reduced LC3-II levels. In contrast, 3-MA did not affect the LC3 levels in Leydig and SC (Figure 7A, lower panels). Moreover, the application of 5 µM of 3-MA induced a reduction in LC3-II levels of male GC and macrophages in a time-dependent manner (Figure 7B). LC3-II levels were remarkably reduced at 24 h after the presence of 3-MA. Notably, 3-MA significantly increased MuV-NP RNA levels in male GC and macrophages at 48 h after the cells were infected with one MOI MuV (Figure 7C, upper panel). In contrast, 3-MA did not significantly affect the MuV-NP RNA levels in Leydig and SC. Results of Western blot analysis showed that 3-MA increased MuV-NP protein levels in male GC and macrophages, but not in Leydig and SC (Figure 7C, lower panel). Immunofluorescence staining confirmed that MuV-NP numbers were increased by 3-MA in macrophages (Figure 7D, left panels) and male GC (right panels). The presence of 3-MA did not affect MuV-NP numbers in Leydig and SC (data not shown). Accordingly, 3-MA significantly increased the MuV titers in the culture media of GC and macrophages but not in that of the Leydig and SC (Figure 7E). Furthermore, 3-MA treatment and MuV infection did not significantly affect the survival of testicular cells (Figure 7F).

## Discussion

The global resurgence of mumps orchitis warrants an in-depth understanding of MuV replication in the testis (29). Unraveling the mechanisms underlying the innate antiviral response against MuV in the testis can aid in the development of preventive and therapeutic strategies for mumps orchitis. The present study investigated MuV replication in testicular cells and cell type-specific antiviral mechanisms. We demonstrated that major testicular cells exhibited different innate antiviral mechanisms against MuV replication.

The present study focused on major testicular cells, including LC, testicular macrophages, Sertoli, and male GC. We used testicular cells from 4-week-old mice based on their ease in isolation from the testis of mice at this particular age. Orchitis occurs in about 40% of pubertal and postpubertal mumps in men and may result in male infertility at a high incidence rate (30). Although mumps orchitis incidence had dramatically declined following the administration of MuV vaccines in children, a recent outbreak of mumps orchitis in postpubertal men is threatening male fertility (31). Studies on MuV replication in testicular cells may facilitate in the elucidation of the mechanisms underlying the development of mumps orchitis. The present study has shown that MuV infects major testicular cells, although its replication in different types of testicular cells varies. The highest rate of MuV replication was observed in SC, followed by LC and macrophages. In contrast, MuV did not replicate in male GC. These observations suggest that testicular cells possess different mechanisms to control MuV replication. The mechanisms by which testicular cells control MuV replication is worthy investigating.

Type 1 IFNs are inducible during viral infection and considered as the primary antiviral response (28). IFN-α and IFN-β induce the expression of numerous antiviral proteins to counteract invading viruses in infected cells and facilitate adaptive immune response against viral infection (8, 32). The major IFN-induced antiviral proteins include ISG15, OAS1, and MX1, which amplify antiviral signaling, degrade viral RNA, and inhibit viral gene transcription, respectively (33). The present study showed that recombinant IFN-β significantly induces antiviral protein expression and inhibits MuV replication in LC, macrophages, and SC. The anti-IFN-β antibodies significantly increased MuV replication in testicular somatic cells, which in turn restricted MuV replication *via* IFN-β production; these findings are in agreement with the results of a previous study, i.e., that type 1 IFN treatment prevents infertility in mumps orchitis patients (34). A high rate of MuV replication was observed in SC compared to LC. This observation may be explained by the fact that SC produce relatively low levels of type 1 IFNs in response to MuV infection compared to LC (12). The present study showed that SC expressed lower levels of antiviral proteins than LC after MuV infection. However, the *in vitro* data may not reflect the *in vivo* situation because IFNs produced by LC should enhance the antiviral response in SC in a paracrine manner. This speculation is supported by the observation that recombinant IFN-β significantly inhibited MuV replication and induced antiviral protein expression in SC. The antiviral response and MuV replication in testicular cells *in vivo* remain to be clarified.

Testicular macrophages display immunosuppressive properties in favor of the immunoprivileged environment in the testis (35, 36). Rat testicular macrophages produce fewer IFNs than LC after infection with Sendai viruses (37). However, the present study showed that MuV replicated at comparable efficiencies in LC and macrophages. This observation implies that testicular macrophages should possess other mechanism to restrict MuV replication in addition to the IFN pathway. Particularly, IFN-β neither induce antiviral protein expression nor inhibited MuV replication in male GC, thereby suggesting that male GC adopted an IFN-independent defense against MuV replication.

Autophagy plays an important role in recycling intracellular components and removing dysfunctional organelles to adapt to starvation stress and maintain cellular homeostasis (38). Recent reports have described the roles of autophagy in regulating spermatogenesis (20, 39). Autophagy is also involved in the cellular defense against microbial infections (40). Autophagosomes may directly uptake and degrade invading viruses intracellularly (41). The present study showed that male GC and macrophages abundantly expressed beclin-1 and LC3, thereby indicating that these cells are well equipped with an autophagic machinery. The 3-MA, which is an inhibitor of autophagy, inhibited LC3-II level. LC3-II is a marker of active autophagy (42). Remarkably, the presence of 3-MA significantly increased MuV replication in male GC and macrophages. These results suggest that autophagy restricts MuV replication in these cell types. On the other hand, both IFN response and autophagy limited MuV replication in testicular macrophages, and autophagy is essential to block MuV replication in male GC. In contrast, beclin-1 and LC3 were faintly expressed in Leydig and SC. Accordingly, 3-MA did not significantly affect MuV replication in Leydig and SC. Therefore, autophagy should not be involved in the control of MuV replication in Leydig and SC. The role of autophagy in restricting MuV replication in the testicular cells needs further corroboration through genetic analysis. In this context, cell-specific knockout of autophagy genes would be an ideal model to confirm the antiviral defense of autophagy in testicular cells.

Recent studies demonstrated that Zika virus infection damages the testis in mice (43, 44). Similar to MuV, Zika virus induced inflammatory cytokine production in testicular somatic cells but not in GC (44). Interestingly, Zika virus was detected in testicular somatic cells and spermatogenic stem cells but not detected in the developing GC. The mechanisms underlying the cell-specific infection of Zika virus remain to be clarified, which should refer to the present study on MuV.

Viral infection in male GC may be transmitted to female partners and fetus, thus leading to virus spreading (45). Therefore, the antiviral defense of male GC are particularly important not only for healthy male fertility but also for limiting the vertical transmission of viruses. Notably, the late stages of male GC are separated from the components of the immune system and antiviral drugs by the blood–testis barrier (BTB). The innate antiviral defense of the male GC behind the BTB should be critical for counteracting viral infection. Moreover, the antiviral response *via* the autophagy pathway does not require the production of pro-inflammatory cytokines, whereas the innate immune responses to microbial pathogens produce numerous pro-inflammatory cytokines. High levels of certain pro-inflammatory cytokines can be harmful to spermatogenesis (46–50). Therefore, autophagy should be a particularly suitable means for the antiviral defense of male GC.

In summary, the present study examined the testicular cell type-specific defense against MuV replication *in vitro*. IFN-β restricts MuV replication in Leydig and SC. Autophagy may play a role in blocking MuV replication in male GC. Testicular macrophages utilize both autophagy and IFN pathways to control MuV replication. These results provide novel insights into the mechanisms underlying the innate defense against MuV replication in testicular cells and suggest that manipulations of cell type-appropriate antiviral response should be considered as preventive and therapeutic approaches against MuV infection in the testis.

## Author Contributions

HW, XZ, and DH designed the project and experiments. HW, XZ, FW, QJ, and LS carried out most of the experiments. HW, MG, and WL isolated the primary cell, and QL isolated and identified MuV. BG, CS, and YC carried out statistical analysis. DH and HW wrote the paper.

## Conflict of Interest Statement

The authors declare that the research was conducted in the absence of any commercial or financial relationships that could be construed as a potential conflict of interest.
